# Association between polypharmacy and the persistence of delirium: a retrospective cohort study

**DOI:** 10.1186/s13030-020-00199-3

**Published:** 2020-10-06

**Authors:** Ken Kurisu, Daisuke Miyabe, Yoshiko Furukawa, Osamu Shibayama, Kazuhiro Yoshiuchi

**Affiliations:** 1 Department of Psychosomatic Medicine, Yokohama Rosai Hospital, Yokohama, Japan; 2 Department of Stress Sciences and Psychosomatic Medicine, Graduate School of Medicine, The University of Tokyo, 7-3-1, Hongo, Bunkyo-ku, Tokyo, 113-8655 Japan

**Keywords:** Classification and regression tree, Delirium, Polypharmacy, Propensity score, Receiver operating characteristic curve

## Abstract

**Background:**

Although the association between polypharmacy and the occurrence of delirium has been well studied, the influence of polypharmacy on the persistence of delirium remains unclear. We aimed to explore the effect of polypharmacy on the persistence of delirium.

**Methods:**

This retrospective cohort study was conducted at a tertiary hospital. The medical records of patients diagnosed with delirium who were referred to the Department of Psychosomatic Medicine were reviewed. Presentation with delirium on day 3 was set as the outcome in this study. We counted the number of drugs prescribed on the date of referral, excluding general infusion fluids, nutritional or electrolytic products, and psychotropics. To define polypharmacy, we developed a classification and regression tree (CART) model and drew a receiver operating characteristic (ROC) curve. The odds ratio (OR) of polypharmacy for the persistence of delirium on day 3 was calculated using a logistic regression model with the propensity score as a covariate.

**Results:**

We reviewed the data of 113 patients. The CART model and ROC curve indicated an optimal polypharmacy cutoff of six drugs. Polypharmacy was significantly associated with the persistence of delirium both before [OR, 3.02; 95% confidence interval (CI), 1.39–6.81; *P* = 0.0062] and after (OR, 3.19; 95% CI, 1.32–8.03; *P* = 0.011) propensity score adjustment.

**Conclusion:**

We discovered an association between polypharmacy and worsening courses of delirium and hypothesize that polypharmacy might be a prognostic factor for delirium.

## Background

Delirium is an acute psychiatric disorder that is caused by physiological and/or pharmacological factors. The body-mind relation is strongly associated with its pathology, making both psychiatric and physiological interventions critical to its management [1].

Polypharmacy is a growing social issue worldwide. Inappropriate prescribing practices mainly contribute to its occurrence [2, 3]. It causes both physiological and psychological problems for patients with multiple morbidities [4, 5].

The association between polypharmacy and the incidence of delirium has been well studied [6–10] and a guideline recommends drug reviews for delirium prevention [11]. However, the influence of polypharmacy on the persistence of delirium has yet to be examined thoroughly. One retrospective study investigated the effect of a drug review on the course of delirium treatment, but did not focus on the effect of the number of drugs [12].

Therefore, we aimed to explore the association between polypharmacy and the persistence of delirium.

## Methods

### Ethics consideration

The institutional review board of Yokohama Rosai Hospital approved the study (No. 31–42) and waived the requirement for informed consent owing to the retrospective design.

### Medical record review

This retrospective cohort study was conducted at Yokohama Rosai Hospital, a tertiary medical center in Japan. We reviewed the medical records of patients (a) who had been suspected to have any psychiatric disorder by physicians in departments other than the Department of Psychosomatic Medicine, (b) who had been referred to the Department of Psychosomatic Medicine from March 2019 to October 2019, (c) who had been diagnosed with delirium, and (d) who had not been under continuous sedation.

Diagnosis was based on the Diagnostic and Statistical Manual of Mental Disorders, Fifth Edition (DSM-5) [13]. It was initially done by senior residents (post-graduate year 3–5), then with supervision by a specialist certified by the Japanese Society of Psychiatric and Neurology (Y.F.).

We defined the first 24 h from 9:00 p.m. on the referral date as day 1, and every subsequent period of 24 h as day N. We reviewed the medical charts to determine whether patients had presented with delirium on day 3, which was denoted as the outcome. Day 3 was selected because a previous study suggested that an outcome evaluation on this day could be used to estimate the treatment effectiveness [14], and this outcome has been widely adopted [15, 16]. Two authors (senior residents K.K. and D.M) reviewed the medical records independently by referring to the prepared manual. The manual included (a) confirmation of the baseline (before referral) level of consciousness, (b) confirmation of satisfaction of the diagnostic criteria of DSM-5 [13], and (c) discussion about disagreements in evaluation of each item of the diagnostic criteria. Production of the manual was supervised by a board member of the Japanese Society of Psychosomatic Medicine (K.Y.). The reproducibility of the evaluation (before discussion about disagreements) was analyzed using the kappa statistic.

The number of drugs prescribed on the referral date was counted, excluding general infusion fluids, nutritional or electrolytic products, and psychotropic agents.

### Statistical analysis

We used the t-test to compare the means of continuous variables, and Fisher’s exact test or the chi-squared test to compare the proportions of categorical variables for patients with and without delirium symptoms on day 3.

We performed the following analyses to determine the cutoff for polypharmacy [17, 18]. First, we developed a classification and regression tree (CART) model in which the maximum tree depth was fixed at one and the Gini index was used as the splitting metric. We considered the splitting value of the root node to be the cutoff value. We also generated a receiver operating characteristic (ROC) curve and calculated the Youden index for each cutoff point. The cutoff that maximized the Youden index was considered the optimal cutoff value.

We calculated the odds ratio (OR) of polypharmacy for the persistence of delirium on day 3 using a logistic regression model with the propensity score as a covariate. Propensity scores were calculated using a logistic regression model based on the following variables: age, sex, the presence of 17 diseases in the updated Charlson comorbidity index (an index to predict mortality based on the presence of comorbid conditions) [19], the Karnofsky Performance Status (an index of physical performance ability) [20] score on admission, and psychotropics (five categories: antipsychotics, benzodiazepine or Z-drugs, ramelteon or suvorexant, antidepressants, and others). The Karnofsky Performance Status was used as an index of severity. Psychotropics were included because of their potential effects on delirium [1, 21]. To confirm the sufficiency of the sample size, we performed a power analysis of the univariate logistic regression model.

All analyses were conducted using R version 4.0.0 (R Foundation for Statistical Computing, Vienna, Austria, 2019) with the packages ‘powerMeditation’ (version 0.3.2), ‘ROCR’ (version 1.0–11), ‘rpart’ (version 4.1–15), and ‘vcd’ (version 1.4–7). A *P*-value < 0.05 was considered statistically significant.

## Results

### Eligible patients

Of the 113 patients reviewed, 64 (57%) did not have symptoms of delirium on day 3. The kappa statistic for a delirium evaluation was 0.66 (95% confidence interval [CI] = 0.53–0.80), which showed a substantial degree of reproducibility. The descriptive data are shown in Table 1. Only the Karnofsky Performance Status score differed significantly between patients with and without symptoms of delirium on day 3.

**Table 1:** Patient characteristics (*N* = 113)

	Delirium (*n* = 49)	No delirium (*n* = 64)	*P*-value
Age (years), mean (SD)	80.76 (11.90)	81.81 (8.25)	0.60^‡^
Male sex, n (%)	28 (57)	34 (53)	0.81^*^
Charlson comorbidity index, mean (SD)	3.14 (2.02)	2.92 (2.35)	0.59^‡^
Karnofsky Performance Status, mean (SD)	39.59 (20.61)	48.44 (25.02)	**0.04**^‡^
Number of drugs, mean (SD)	7.04 (3.26)	6.14 (3.95)	0.19^‡^
Number of drugs with psychotropics, mean (SD)	10.02 (3.78)	8.66 (4.11)	0.07^‡^
Psychotropics use, n (%)			
Antipsychotics	33 (67)	39 (61)	0.61^*^
Benzodiazepine or Z-drugs	12 (24)	13 (20)	0.76^*^
Ramelteon or suvorexant	41 (84)	55 (86)	0.95^*^
Antidepressant	3 (6)	2 (3)	0.65^†^
Other drugs	19 (39)	18 (28)	0.32^*^

Other drugs included anti-dementia drugs, valproic acid, Yokukansan

^*^, Chi-squared test; ^†^, Fisher’s exact test; ^‡^, Student’s *t*-test

### Number of drugs and persistence of delirium

**Fig. 1:**
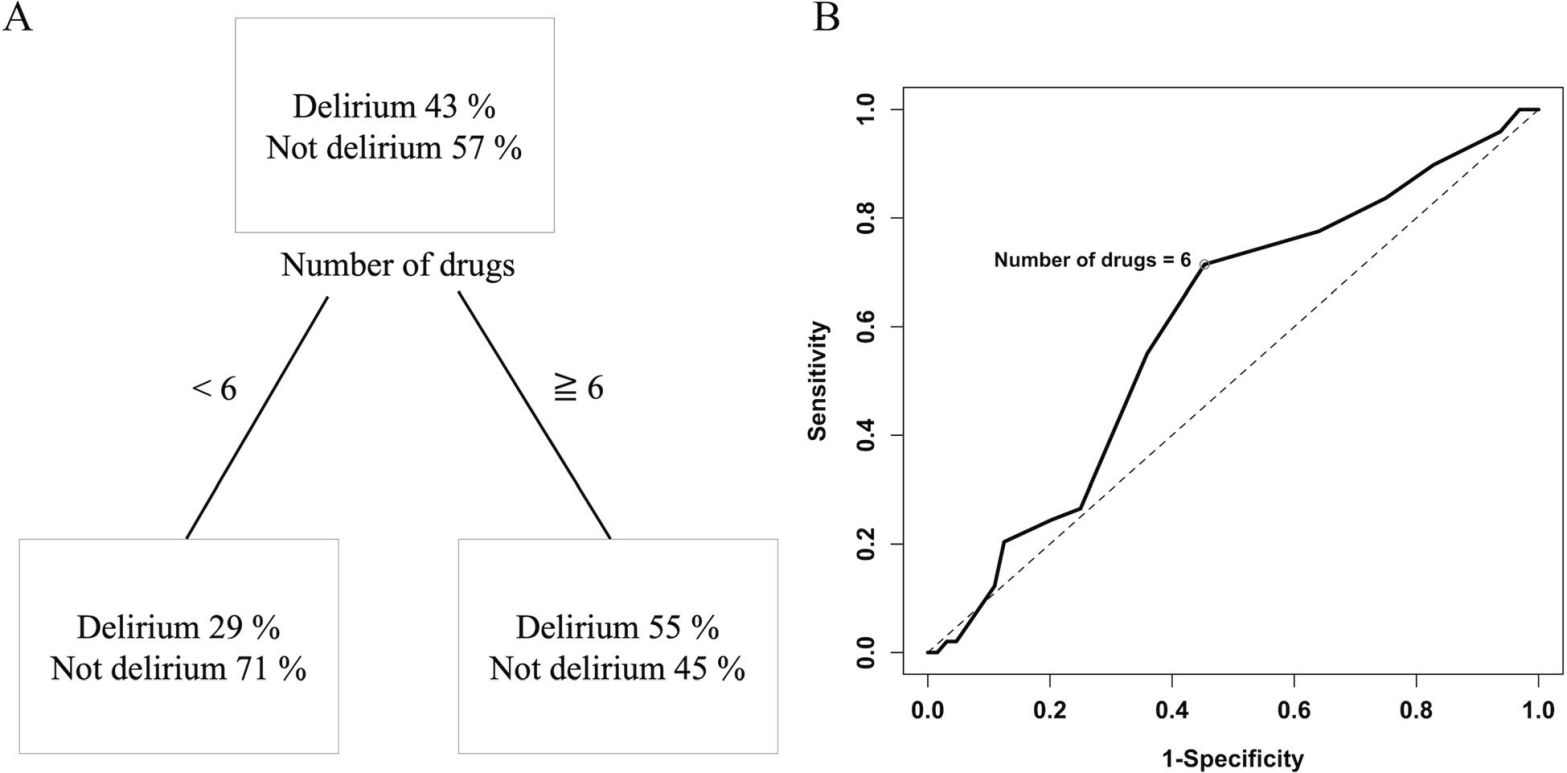
Analysis of cutoffs related to the persistence of delirium on day 3. **a** A classification and regression tree model used to determine the cutoff point for the number of drugs associated with the persistence of delirium on day 3. The root node splitting value was six. **b** A receiver operating characteristic curve used to determine the cutoff number of drugs associated with the persistence of delirium on day 3. The maximum Youden index was calculated at a cutoff of six drugs

The splitting value of the root node in the CART model was six, and this number also maximized the Youden index in the ROC curve (Fig. 1). Therefore, we defined polypharmacy as the use of six or more drugs. Sixty-four (57%) patients in the cohort met this definition of polypharmacy.

As shown in Table 2, polypharmacy was significantly associated with the persistence of delirium on day 3 in the absence (OR, 3.02; 95% CI, 1.39–6.81; *P* = 0.0062) and presence of the propensity score as a covariate (OR, 3.19; 95% CI, 1.32–8.03; *P* = 0.011). The C-statistic of the propensity score model was 0.767. The power of the significance of polypharmacy in the univariate model was 0.808, suggesting that the sample size was sufficient for the study.

**Table 2:** Odds ratios of polypharmacy for the persistence of delirium on day 3

	OR	95% CI	*P*-value
Without propensity score	3.02	1.39–6.81	**0.0062**
With propensity score as a covariate	3.19	1.32–8.03	**0.011**

*OR* odds ratio, *CI* confidence interval

## Discussion

The present study assessed the association between polypharmacy and the persistence of delirium and showed that the use of six or more drugs was associated with the persistence of delirium.

The results suggest that polypharmacy may not only induce delirium, which is known [6–8], but that it may also worsen the course of delirium treatment. Because delirium occurs due to a body-mind relation and polypharmacy is a social issue, the results are an example of bio-psycho-social pathology [22]. The results support the claims of a previous study that reported the effectiveness of a drug review on the course of delirium treatment [12].

Additionally, the cutoff of six drugs, which was calculated based on the ROC curve and CART model, was congruent with the values reported by previous studies for the association of polypharmacy with adverse effects [5–10]. The incidence of drug–drug interactions, which is considered to be associated with delirium [23], may increase drastically when the number of drugs exceeds six.

This study had several limitations. First, because the study was conducted at a single institution, sampling bias could exist. Second, the present study only included patients who were suspected by non-expert medical staff to have psychiatric disorder. Because the rate of undetected delirium is extremely high [24], the generalizability of the results might be limited. Third, the outcome was evaluated based on a retrospective chart review, and therefore its accuracy could not be fully validated. Fourth, the outcome evaluation was based on DSM-5 [13] and not on a structured clinical interview; therefore, it would not be robust. Fifth, although both the diagnosis and the outcome evaluation were supervised by trained specialists, senior residents mainly performed the work. Sixth, there was no precise criteria to resolve disagreements about the outcome evaluation. Seventh, the propensity score model did not include the severity of delirium (e.g., severity score measured using the Delirium Rating Scale, Revised-98), comorbid diseases that were not listed in the Charlson comorbidity index [19], or laboratory test data. Finally, the contents of the drugs were not considered in the analyses.

## Conclusions

We discovered an association between polypharmacy and a worsening course of delirium in this pilot study. Therefore, we hypothesize that polypharmacy might be a prognostic factor for delirium. However, due to the limitations, further studies will be required to validate the present finding.

## Data Availability

The datasets and source codes are available from the corresponding author on reasonable request.

## Abbreviations

DSM-5: Diagnostic and Statistical Manual of Mental Disorders, Fifth Edition
CART: Classification and regression tree
ROC: Receiver operating characteristic
OR: Odds ratio
CI: Confidence interval
